# Prophylactic Left Internal Mammary Artery Graft In Mildly-Stenosed Coronary Lesions. Still An Open Discussion

**DOI:** 10.5935/abc.20160032

**Published:** 2016-03

**Authors:** Paulo Roberto B. Evora, Livia Arcêncio, André Schmidit, Alfredo José Rodrigues

**Affiliations:** 1 Departamento de Cirurgia e Anatomia, Faculdade de Medicina de Ribeirão Preto, Universidade de São Paulo, Ribeirão Preto, SP - Brazil; 2 Departamento de Clínica Médica, Faculdade de Medicina de Ribeirão Preto, Universidade de São Paulo, Ribeirão Preto, SP - Brazil

**Keywords:** Mammary Arteries/transplantation, Coronary Stenosis, Coronary Artery Disease

## Introduction

The main advantages of CABG with the left internal mammary artery (LIMA) have been well established since the 80s and can be enumerated as follows.^1^

1. Increased long-term patency, undoubtedly demonstrated;
2. Atherosclerosis resistance based on the endothelial production of nitric oxide and prostacyclin;
3. Contrary to the venous bypass, it has been used not only for pedicle grafts as well as for free grafts;
4. Improved clinical outcomes (various studies have shown the influence of LIMA graft on the recurrence of angina, nonfatal myocardial infarction, and favorable survival, and;
5. Less need for reoperations (a 2-fold lower incidence of reoperations has shown in patients without LIMA graft compared to the left anterior descending coronary artery (LAD).

It has been well established that coronary venous grafts in arteries with moderate atherosclerotic lesions (<70%) had early occlusion mainly due to flow competition with the native coronary circulation. Otherwise, to graft a moderately stenosed coronary vessel with LIMA remains debatable, keeping the question by Hayward and colleagues open: "Should all moderate coronary lesions be grafted during primary coronary bypass surgery?".^2^ However, controversy exists whether LIMAs should be used to bypass coronary arteries with noncritical stenoses.^3^

### Left internal mammary flow

Doubts about the quality of LIMA flow began to fade in the late 1970s. However, in the 1980s, numerous studies demonstrated the ability to LIMA to dilate or decrease its diameter according to the myocardial needs, demonstrating the dynamic nature of its luminal diameter.

Excluding surgical problems (damage during harvesting and mobilization, spasm, inflammation, or a steal phenomenon arising from a large undivided proximal LIMA branches), LIMA graft failure in coronary artery bypass grafting (CABG) is mostly considered to be a result of competitive flow (CF) from the native coronary artery, limiting future revascularization options particularly in young patients.

As time goes by, the controversies remain "alive", emphasizing that experimental studies, concerning the "prophylactic" use of LIMA grafts for moderate coronary obstructions, demonstrate and keep controversial results. Results from acute experiments have indicated that competitive flow from a fully patent native artery did not abolish LIMA graft flow. The chronic experiments results demonstrate that even after 2 months of maximal chronic flow competition from a fully patent native artery, LIMA graft flow was maintained above *in situ* levels, and a recruitable flow reserve could be demonstrated when the native vessel was occluded. These data suggest that LIMA grafts are dynamic and may remain patent despite significant residual flow in the native vessel.^4^ LIMA graft patency decreases as coronary artery competitive flow increases. However, the effect of competitive flow on LIMA graft patency is mild, and no degree of proximal coronary stenosis led to declining LIMA patency. This finding suggests that LIMA should not be avoided when bypassing coronary arteries with moderate degrees of stenoses.^3^

### The concept of "prophylactic" grafting

The concept of "prophylactic" grafting is an interesting one. It is particularly appealing in those patients with concomitant severe medical illnesses in whom coronary bypass reoperation procedures would pose a considerable risk. In these patients, grafting minimally diseased vessels that have the potential to become hemodynamically significant with time might be a reasonable option, which might afford the patient a longer disease-free interval. Twenty years ago Lust and colleagues wrote that in the future, with more data, prophylactic grafting might be considered, but at that time, they did not believe in making that statement.^5^ Nowadays, it is possible to conclude that the subject remains an open discussion and deserves actions to become the consensus in our guidelines.

The decision to graft or leave a moderately stenosed vessel during a cardiac surgical procedure depends on some calculations by the surgeon. In clinical practice, the balance of these estimations of the future of both the lesion and any graft placed to that territory must be weighed against other surgical considerations, such as the availability of conduit, the number of grafts, and other operative procedures needed, such as valvular or aortic repair. Faced with a moderate lesion, the surgeon might commonly choose between leaving it alone or placing a saphenous vein bypass. The greater risk of progression of left-sided moderate lesions and high graft patency rates when bypassed, suggests that the balance of clinical judgment lies in favor of grafting moderate left-sided lesions. Data from postoperative angiography in predominantly asymptomatic patients receiving contemporary secondary prevention therapies suggests that bypass grafting best treats moderate lesions in the left coronary system during multivessel revascularization. However, right-sided lesions may reasonably be left alone because they are unlikely to progress and are not likely to require subsequent revascularization. These data may assist coronary surgeons in a joint clinical dilemma.^2^

The internal mammary graft is a physiologically active conduit that is dependent on flow dynamics. One emblematic reported case evidenced that competitive flow through the nonobstructive native LAD in combination with an impedance of flow through the LIMA due to a severe lesion in the LAD distal to the anastomosis led to a functionally occluded LIMA. When the obstruction in the proximal LAD progressed, and the distal obstruction was successfully angioplastied, the LIMA flow dynamics improved, allowing for its dilatation and restoration of patency. Therefore, an angiographically occluded internal mammary graft may be only functionally occluded and reversible even when the occlusion is demonstrated several days apart.^5^

The association between competitive flow and hemodynamics, as a kind of consensus, is still unclear. There is scarce literature focusing experimentally or clinically on this area. About supplemental vein grafting for LIMA hypoperfusion, an experimental study in dogs compared LIMA flow in different settings. The results showed that the vein graft placed distally or proximally limits LIMA flow and LIMA contribution to distal perfusion both in the resting heart and during the increased myocardial oxygen demand.^6^ Clinically, Kawamura et al.^7^ studied the effect of competitive flow on patency rate of the internal thoracic artery to the left anterior descending artery bypass from the concomitant saphenous vein (SV) graft in the left coronary artery, based on 313 patients who had two bypasses to the left coronary artery including in situ LIMA-LAD graft. It was also concluded that competitive flow from SV graft could play a major role in occlusion of the in situ arterial graft.

Even though the overall patency rate of IMA grafts is high, the present data indicate that the long-term patency rate of IMA grafts is low when the recipient's vessel is only moderately stenosed. Basically, these findings imply the decision to use an IMA should be carefully considered in light of the hemodynamic severity of the stenosis in the recipient's vessel. This might avoid the inappropriate use of an IMA as a graft to a recipient artery that does not need to be revascularized.^8^

## Conclusion

In conclusion, LIMA has the capacity of flow adaptation according to the myocardial metabolic necessities. Also, it has a "hibernating" capacity, protecting against coronary artery disease. Therefore, all moderate coronary lesions should be LIMA grafted during primary coronary bypass surgery.^9^ On the other hand, the LIMA graft occlusion due to flow competition prevents the possibility of future use in an eventual CABG reoperation. Nowadays, the idea of a prophylactic LIMA on LAD in mild-stenosed vessels is not confirmed yet by clinical evidence.
